# Percutaneous Inferior Extensor Retinaculum Augmentation Technique for Chronic Ankle Instability

**DOI:** 10.1111/os.13248

**Published:** 2022-04-18

**Authors:** Shengxuan Cao, Chen Wang, Xu Wang, Xin Ma

**Affiliations:** ^1^ Department of Orthopedics Huashan Hospital, Fudan University Shanghai China

**Keywords:** chronic ankle instability, complication, functional outcome, inferior extensor retinaculum, modified Brostrom–Gould procedure

## Abstract

**Objective:**

To specify indications and contraindications of the modified percutaneous inferior extensor retinaculum augmentation (PIERA) technique for chronic ankle instability cases, and to introduce technique details and report surgical outcomes and complications.

**Methods:**

The PIERA technique was performed on seven patients with chronic ankle instability (four females and three males, 36.4 ± 15.1 years of age, and course of symptoms of 33.7 ± 8.8 months) from June to October 2018 in this retrospective study of case series. All patients demonstrated attenuated ligamentous tissue quality, which was confirmed using preoperative ankle MRI. IER were drew up to the distal fibula using suture anchors with the ankle in neutral position for all cases, to engage the entire IER in reconstructing the stability of the ankle. Patients were assessed using American Orthopaedic Foot and Ankle Society Ankle‐Hindfoot (AOFAS) score and Cumberland Ankle Instability Tool (CAIT) scores pre‐ and postoperatively at the last follow‐up examination. Preoperative and postoperative outcome scores of patients were compared using paired *t*‐test. A *p* value of less than 0.05 was regarded statistically significant.

**Results:**

Mean follow‐up duration was 16.7 ± 1.6 months. The mean AOFAS score significantly improved from 66.9 ± 11.2 preoperatively to 93.7 ± 8.5 postoperatively (*P* = 0.001). Mean CAIT score significantly improved from 13.1 ± 4.7 preoperatively to 26.3 ± 1.8 postoperatively (*P* = 0.001). Patients did not report any wound healing problem, numbness, swelling, or instability at the last follow‐up examination, except for one patient who reported pain and minimal stiffness, and presented an AOFAS score of less than 80 and a CAIT score below 24. All patients returned to at least recreational sport activity level.

**Conclusion:**

The PIERA technique can improve the functional outcomes of patients with chronic ankle instability with few complications.

## Graphical abstract

We presented the percutaneous inferior extensor retinaculum augmentation (PIERA) technique for chronic ankle instability in this study. Inferior extensor retinaculum (IER) were drawn up to the distal fibula using suture anchors with the ankle in neutral position to engage the entire inferior extensor retinaculum in reconstructing the stability of the ankle. This technique is convenient to perform and appropriate for chronic ankle instability cases with attenuated ligamentous tissue quality.

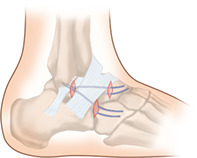

## Introduction

Lateral ankle ligamentous injury caused by ankle sprains may lead to chronic ankle instability. Patients with chronic ankle instability suffer from ankle pain, swelling, recurrent ankle sprain, giving way, and feeling of instability. Brostrom reported anterior talofibular ligament (ATFL) and calcaneofibular ligament (CFL) repair technique for chronic ankle instability. Gould^1^ modified this technique with inferior extensor retinaculum (IER) augmentation. The modified Brostrom–Gould procedure has been the gold standard for treating chronic ankle instability, with solid biomechanical and clinical evidence reported in several studies^2^, ^3^. The minimally invasive arthroscopic Brostrom technique has been widely used, and biomechanically and clinically equal to the open Brostrom technique^4^, ^5^, ^6^.

Although the modified Brostrom–Gould procedure with lateral ankle ligament repair and IER augmentation yields satisfactory results, several modifications of this technique are more convenient and equally effective for chronic ankle instability. Takao *et al*.^7^ proposed the arthroscopic ATFL repair technique. A study involving this technique reported a mean AOFAS score of over 90 at last follow‐up examination^8^. Different from the traditional modified Brostrom–Gould procedure, the arthroscopic ATFL repair technique leaves CFL unrepaired without IER augmentation. In patients who are not candidates for standard repair, arthroscopic ATFL repair technique is not recommended^9^.

By comparison, Acevedo *et al*.^10^ reported that IER is typically used rather than ATFL for reconstruction of ankle stability, which has achieved satisfactory follow‐up data. Sutures pass through the IER and pull the IER to fibular periosteum in this technique. The use of IER in the operative treatment of chronic ankle instability is common from classic modified Brostrom–Gould procedure to newly introduced techniques^1^, ^10^, ^11^. IER augmentation is suitable for cases with attenuated ligamentous quality. In these cases, IER may provide sufficient stability, and absence of ATFL remnant does not affect the clinical outcomes^11^.

However, the minimally invasive technique of Acevedo *et al*. is highly dependent on expert experience in locating the precise area of the IER to pass sutures due to the high risk in causing iatrogenic injury of nerves and vessels. Surgery‐related complications occur in 7.92% of ankles treated with open surgery and 15.27% of ankles treated arthroscopically^12^. Hence, a convenient technique with direct exposure of pertinent structures is necessary to reduce the risk of iatrogenic injury.

We introduced a modified percutaneous inferior extensor retinaculum augmentation (PIERA) technique based on the technique of Acevedo *et al*. for chronic ankle instability in which sutures pull the entire IER to the anchor located on the fibula in this study. This technique can presumably provide adequate strength using IER augmentation^13^, ^14^, especially suitable for chronic ankle instability cases with attenuated ligamentous quality. This study aims to: (i) specify indications and contraindications of the PIERA technique for chronic ankle instability cases; and (ii) introduce technique details and report surgical outcomes and complications.

## Methods and Materials

### Patients

This study was approved by the review board of our institution. Informed consent forms were collected from all participants that satisfy the following inclusion criteria: (i) Patients with chronic ankle instability with attenuated ligamentous quality, characterized as thin or absent confirmed through preoperative MRI^11^, ^15^, complaints of recurrent ankle sprain (at least two sprains in the same ankle), giving way (more than twice in the past 6 months), or feeling of instability in the previously injured ankle during daily life activities after an initial ankle sprain; and (ii) intervention used was the PIERA technique after unsuccessfully treating with a rehabilitation protocol for at least 3 months.

Exclusion criteria of this study were patients with: (i) a history of fracture or surgery in either lower extremity, or concomitant ankle arthritis; (ii) morbid obesity, generalized joint laxity, high demand in sports, or failed previous ankle stabilization procedure; and (iii) hindfoot varus alignment treated with calcaneal osteotomy.

These patients underwent the PIERA technique after unsuccessfully treating with a rehabilitation protocol for at least 3 months. All surgeries were performed by the same senior orthopaedic surgeon (XM). Patients were assessed using American Orthopaedic Foot and Ankle Society Ankle‐Hindfoot (AOFAS score) and Cumberland Ankle Instability Tool (CAIT score) scores preoperatively and postoperatively.

### Surgical Procedure

#### Arthroscopic Evaluation

Patients under general anesthesia laid supine on an operation table, with a tourniquet placed on the root of the thigh. Towel bumps were placed under the ankle and ipsilateral hip for a slight internally rotated lower limb and sufficient distraction.

Anatomic landmarks, including superficial peroneal nerve, distal fibula, and peroneal tendon, were located preoperatively. The IER is approximately 1.5‐cm distal from the fibula inside a “safe zone” contoured by the three anatomic landmarks^10^. Routine anteromedial and anterolateral portals were created in front of the ankle joint. Intra‐articular lesions were confirmed and addressed arthroscopically while debriding the impingement tissue at the lateral gutter. Footprints of lateral ankle ligaments were confirmed and debrided with a shaver to facilitate the following anchor placement.

#### Inferior Extensor Retinaculum Augmentation

**Fig. 1:**
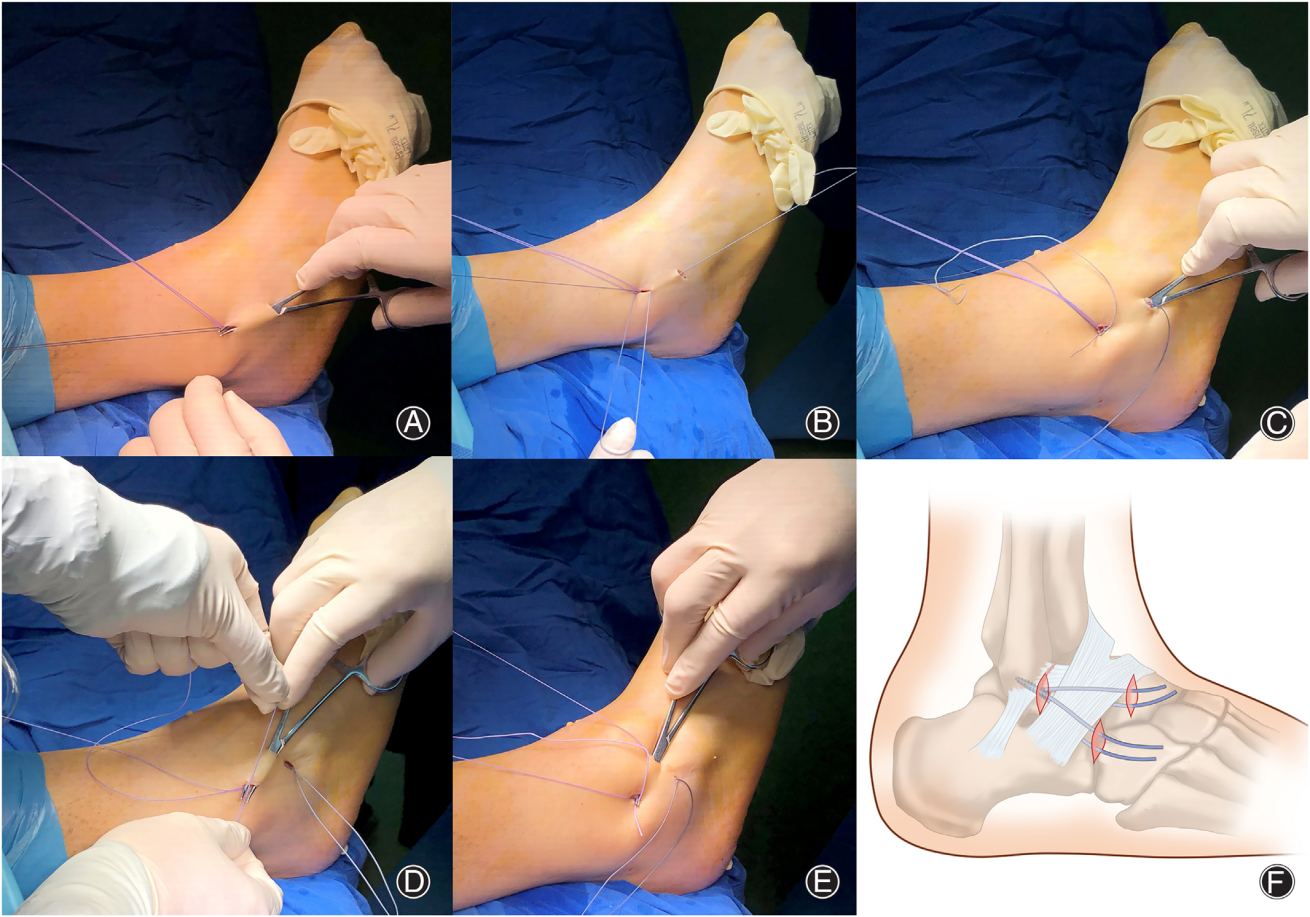
(A) Hemostat is placed through the third portal beneath the subcutaneous tissue and above the IER and comes out through the anterolateral portal in a 26‐year‐old male patient. (B) Hemostat grabs one of the four suture limbs going beneath the subcutaneous tissue and above the IER to guide the strand out of the third portal. (C) Hemostat is placed through the third portal beneath the IER. (D) Passage of the third suture limb from the anterolateral portal to the fourth portal is achieved with the hemostat above the IER. (E) Passage of the fourth suture limb from the anterolateral portal to the fourth portal is achieved with the hemostat beneath the IER. (F) The schematic illustration of this technique where IER was drawn up to the distal fibula using suture anchors with the ankle in neutral position to engage the entire IER in reconstructing the stability of the ankle.

A suture anchor was predrilled and inserted in the midpoint of ATFL and CFL footprints at approximately 1 cm from the distal tip of fibula under the assistance of arthroscopy. The anchor was impacted and confirmed stable by performing a gentle tug on suture limbs. Four strands of suture limbs were retrieved through the anterolateral portal (Figure 1A).

Two 5‐mm‐long extra portals, distal upper and lower ones, were created at the distal border of IER and inside the “safe zone”^10^. Extra portals created at the distal border of IER can be retracted or extended to improve exposure of the distal border of IER. A hemostat was placed through the third portal, beneath the subcutaneous tissue, and above the IER, that came out through the anterolateral portal (Figure 1A). The hemostat grabbed one of the four suture limbs going beneath the subcutaneous tissue and above the IER to guide one strand out of the distal lower portal (Figure 1B).

Similarly, we used hemostat again, and placed it through the distal lower portal beneath the IER (Figure 1C). The hemostat grabbed another one of the four suture limbs going beneath the IER to guide the suture limb out of the third portal. Passage of the two remaining suture limbs from the anterolateral portal to the fourth portal above (Figure 1D) and beneath (Figure 1E) the IER, respectively, was achieved with a hemostat in the same fashion.

Sutures above and beneath the IER were tied in a surgeon or arthroscopic slip knot and then followed by a square knot when the foot was held in neutral position. Remaining sutures above and beneath the IER were tied in the same way (Figure 1F). Hindfoot stability was assessed through manual anterior drawer and talar tilt tests. Four portals were closed with interrupted stitches using 4–0 non‐absorbable sutures.

### Postoperative Protocol

Patients were immobilized in a short‐leg plaster cast for 0–3 weeks postoperatively. Weightbearing is disallowed. Sutures were removed at 10–14 days.

From 3–6 weeks postoperatively, patients were allowed partial to full weightbearing in walking boots at 3–6 weeks postoperatively and gentle range of motion exercises were begun while avoiding excessive ankle inversion.

Patients began walking and jogging without orthotics and formal physical therapy that incorporates heel rise and wobble board exercise at 6–12 weeks postoperatively. The majority of patients returned to pre‐injury activity level at 6 months postoperatively.

### Outcome Measures

Patients were assessed using AOFAS and CAIT scores pre‐ and postoperatively at the last follow‐up examination.

#### American Orthopaedic Foot and Ankle Society Ankle‐Hindfoot Score

The AOFAS score, which is a maximum of 100 points (best possible outcome), was used to evaluate postoperative recovery of the ankle‐hindfoot function. The AOFAS score system mainly includes aspects of pain, function (activity limitations, maximum waking distance, walking surfaces, gait abnormality, sagittal motion, hindfoot motion, and ankle‐hindfoot stability), and alignment. A total score of <80 is considered a poor outcome, while a total score of 80–100 is considered good to excellent.

#### Cumberland Ankle Instability Tool Score

The CAIT score was used to evaluate patient‐report ankle instability. The CAIT score system mainly includes nine aspects of pain, function (level of ankle instability during sports, sharp turns, going down the stairs, standing on one leg, hopping, running, and roll‐over on ankles), and recovery rate. The score can achieve a maximum of 30 points (best possible outcome). A total score of <24 indicates ankle instability, while a total score of 24–30 represents a stable ankle.

### Statistical Analysis

All statistical analyses were performed with SPSS (Version 19.0, SPSS Inc., USA). Outcome scores were compared using the paired t‐test. A *p* value less than 0.05 was considered statistically significant.

## Results

### Intraoperative Results

**Fig. 2:**
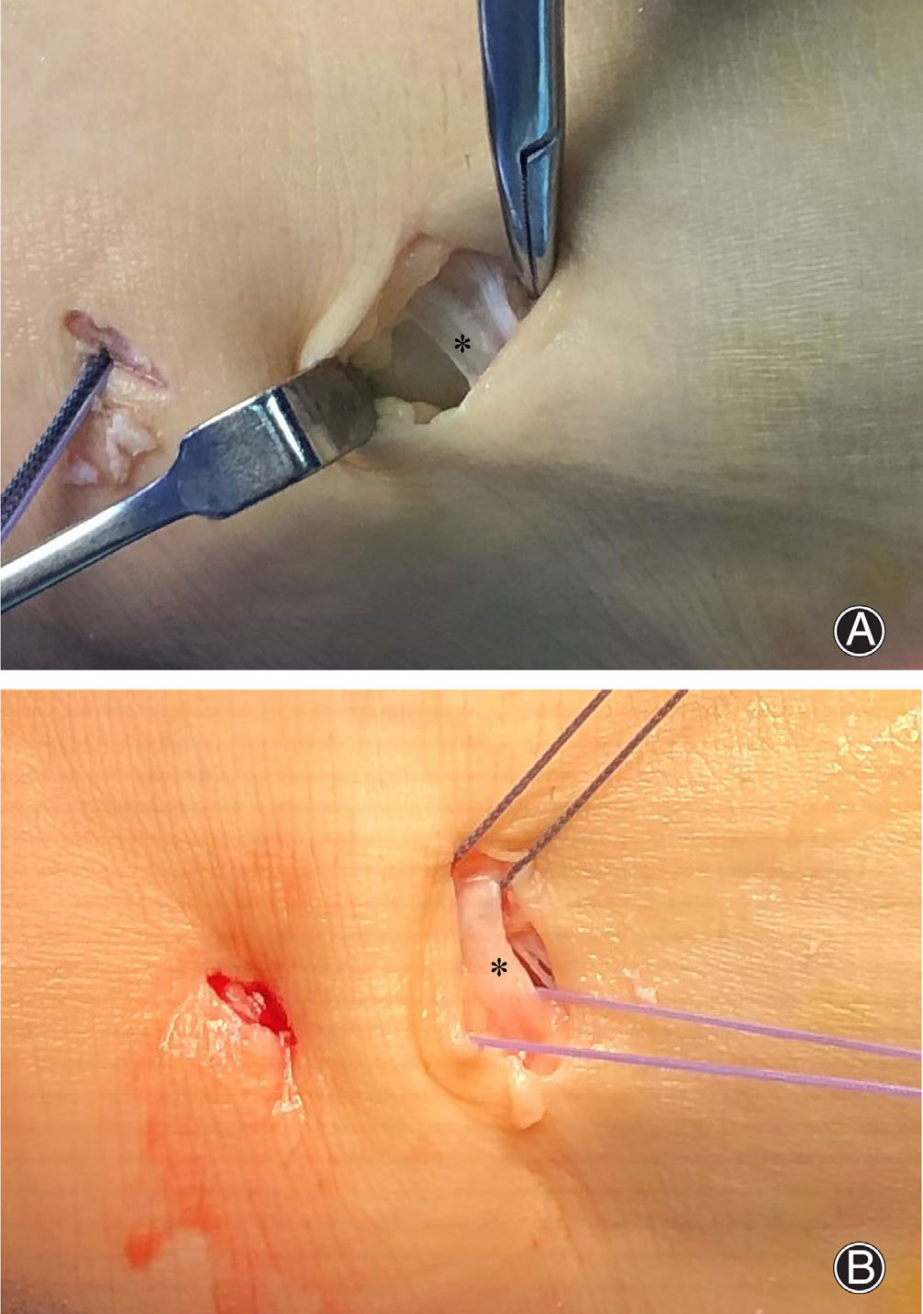
(A) Extra portals created at the distal border of the IER are extended to improve the exposure of the distal border of the IER in a 33‐year‐old male patient. (B) Two sutures go above the IER and two sutures go beneath the IER. *, IER.

We performed this technique on seven patients (patient information is listed in Table 1, four females and three males, 36.4 ± 15.1 years of age, and course of symptoms of 33.7 ± 8.8 months) from June to October 2018. All patients underwent arthroscopic examination, debridement, and PIERA procedure. Three patients had loose body removal, while two patients had microfracture on talus. Extra portals created at the distal border of IER were extended to improve exposure of the distal border of IER in two patients (Figure 2). IER was drawn up to the distal fibula using suture anchors with the ankle in the neutral position for all patients.

**Table 1:** Information of included patients

No.	Gender	Age (years)	Course of symptoms (months)	Additional procedures	Follow‐up duration (months)	Preop AOFAS	Postop AOFAS	Preop CAIT	Postop CAIT
1	Female	62	47	Subtalar debridement	19	67	98	5	26
2	Male	33	25	Microfracture	19	87	95	13	27
3	Female	18	36	Loose body removal	16	67	100	9	28
4	Female	28	26	Subtalar debridement + loose body removal	16	57	90	15	25
5	Male	38	40	Loose body removal	16	75	97	19	27
6	Male	26	38	Microfracture	16	55	100	16	28
7	Female	50	24	Subtalar debridement	15	60	76	15	23

AOFAS, American Orthopaedic Foot and Ankle Society; CAIT score, Cumberland Ankle Instability Tool.

#### American Orthopedic Foot and Ankle Society Ankle‐Hindfoot Score

Mean follow‐up duration was 16.7 months. The mean AOFAS score significantly improved from 66.9 ± 11.2 preoperatively to 93.7 ± 8.5 postoperatively (*P* = 0.001). Only one patient demonstrated an AOFAS score of less than 80. The remaining patients reported satisfactory results and returned to pre‐injury level of activity within 6 months.

#### Cumberland Ankle Instability Tool Score

The mean CAIT score significantly improved from 13.1 ± 4.7 preoperatively to 26.3 ± 1.8 postoperatively (*P* = 0.001). Only one patient demonstrated a CAIT score of less than 24 at the last follow‐up examination.

#### Complications

Patients demonstrated no wound healing problem, numbness, swelling, or instability at the last follow‐up examination. Only one patient reported pain, minimal stiffness. This patient was treated with postoperative rehabilitation engaging wobble board and resistance tube training for 3 months and reported relief for pain and stiffness.

## Discussion

### Indications

The main indication for this technique is chronic ankle instability with attenuated ligamentous quality. We recruited patients with a mean course of symptoms of 33.7 ± 8.8 months in this study. The attenuated quality of ATFL of patients fails to provide sufficient strength in restoring ankle stability. IER is a Y‐shaped structure with normally three separated footprints inside the subtalar joint, commonly used for ankle stability reconstruction^16^, to provide strength physiologically in restraining the excessive subtalar inversion^17^. IER augmentation is similar to the anatomic repair of ATFL and CFL given that ATFL and CFL show a fiber connection between their footprints^18^.

### Surgical Outcomes

The PIERA technique in our preliminary report obtained satisfactory results in most patients. All patients returned to at least recreational sport activity level. An average AOFAS score of 93.7 ± 8.5 is comparable to classic modified Brostrom–Gould procedure, Takao's technique^8^, and Acevedo's technique^10^, which suggested that regular ankle stability can be reconstructed either with lateral ankle ligament alone, IER alone, or both lateral ankle ligament and IER. However, chronic ankle instability cases with attenuated ligamentous tissue quality require strong structures to restore lateral ankle stability^9^, ^11^, which is the rationale of the PIERA technique.

### Technique Details

**Fig. 3:**
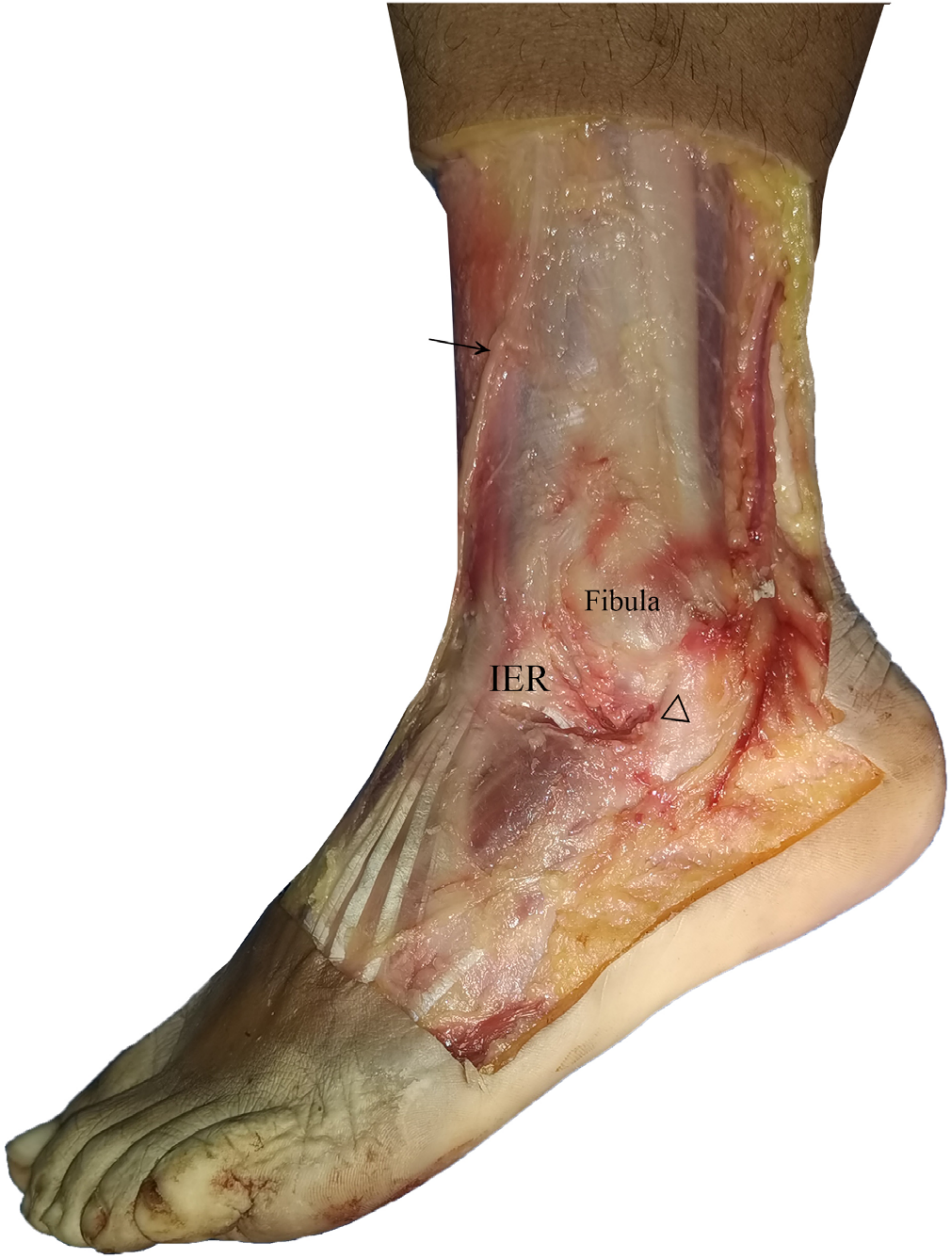
IER, superficial peroneal nerve, fibula, and peroneal tendon are demonstrated on a cadaveric specimen. →, superficial peroneal nerve; △, peroneal tendon.

**Fig. 4:**
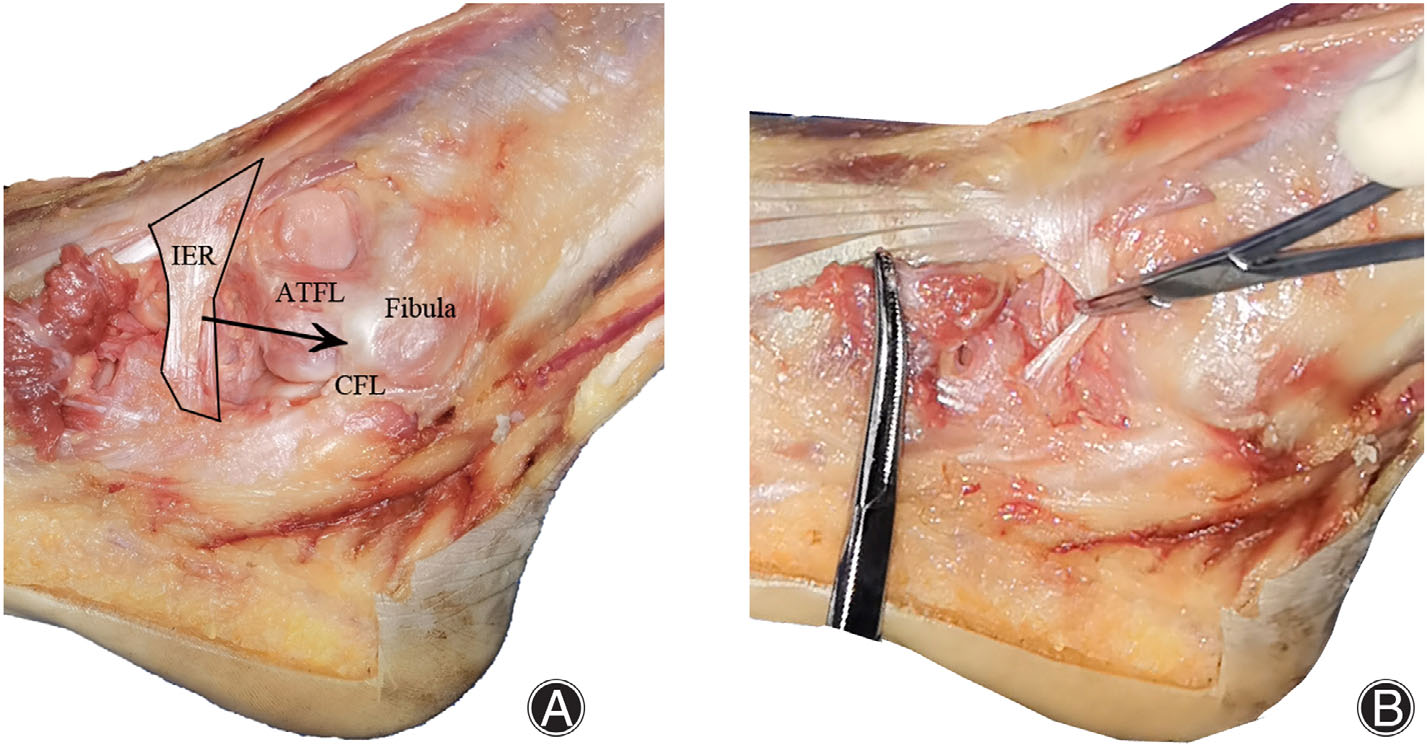
Entire IER is drawn up to the distal fibula with a hemostat in a cadaveric specimen to provide acceptable strength.

The PIERA technique resembles the method described by Acevedo *et al*. because both techniques generally use IER for the reconstruction of ankle stability without emphasis on the repair of lateral ankle ligaments^10^. However, the current technique uses hemostats to pass sutures instead of sharp‐tipped suture passers and reduces iatrogenic injury of adjacent nerves and vessels. This technique also pulls the entire IER to the fibula rather than half of the IER in Acevedo's technique and presumably provides more strength to prevent excessive inversion of the hindfoot. Hence, this technique is a meaningful adaptation of previous techniques that reduces the risk of iatrogenic nerve or vessel injury (Figure 3, 4).

Some authors are concerned that the part of IER used to reconstruct ankle stability fails to provide adequate strength^19^. We used two methods to evaluate the quality of the IER during operation. First, we evaluated the quality of the IER with a gentle tug on suture limbs to assess the strain of the tissue before suture limbs are tied and after four suture limbs of the suture anchor were passed above and beneath the IER. Second, extra portals created at the distal border of IER were extended to improve the exposure of the distal border of IER and allow the direct visual evaluation of the integrity in two patients.

### Limitations

The PIERA technique presents the following limitations. First, in certain patients, the IER is far from the fibula and excessively tight for pulling toward the fibula. This technique may be inappropriate to perform among these patients. A study reported that only 30% of patients were feasible for IER augmentation^20^. Other authors highlighted that the part of IER used to reconstruct ankle stability fails to provide adequate strength, especially in people with an X‐shaped IER^19^. However, these cases were seldom encountered in clinical practice in our institute (Figs 3 and 4). Furthermore, the suture anchor was sufficiently strong to grab the IER and protect the hindfoot from excessive inversion. This outcome was proven using a postoperative manual stress test. Second, the inclusion of seven patients in the current study limits the generalization of the conclusion. Further investigations with validated outcome measures and additional cases are necessary to compare this procedure with other lateral ankle stabilization methods.

### Conclusion

The PIERA technique characterized by ease of use with one suture anchor for chronic ankle instability cases with attenuated ligamentous tissue quality was described in this study. The results revealed that this technique is a convenient and effective procedure that can be used as an alternative treatment for chronic ankle instability cases with attenuated ligamentous tissue quality.

### Authorship Declaration

All authors listed meet the authorship criteria according to the latest guidelines of the International Committee of Medical Journal Editors. All authors are in agreement with the manuscript.
